# Hepatitis B surface antigen reverse seroconversion after hematopoietic stem cell transplantation according to the baseline serological marker levels and vaccination status: a single-center database analysis

**DOI:** 10.1007/s44313-024-00035-5

**Published:** 2024-10-16

**Authors:** Soo Young Kang, Heejoo Ko, Raeseok Lee, Sung-Soo Park, Seunghoon Han

**Affiliations:** 1 Department of Pharmacology, College of Medicine, The Catholic University of Korea, Seoul, Republic of Korea; 2 Division of Infectious Diseases, Department of Internal Medicine, College of Medicine, The Catholic University of Korea, Seoul, Republic of Korea; 3 Division of Hematology, Seoul St. Mary’s Hematology Hospital, College of Medicine, The Catholic University of Korea, Seoul, Republic of Korea; 4 Department of Clinical Pharmacology and Therapeutics, Seoul St. Mary’s Hospital, College of Medicine, The Catholic University of Korea, Seoul, Republic of Korea

**Keywords:** Hematopoietic Stem Cell Transplantation, Hepatitis B Prophylaxis, Reverse Seroconversion, Retrospective; Vaccination

## Abstract

**Purpose:**

Hepatitis B is a major prognostic factor after hematopoietic stem cell transplantation (HSCT). Currently, no consensus exists regarding the management of various scenarios that can lead to reverse seroconversion of the hepatitis B surface antigen (HBsAg-RS). This study focused on HBsAg-RS, which serves as an indicator of active hepatitis, and aimed to obtain exploratory information on the associated patient and treatment factors.

**Methods:**

This single-center retrospective study utilized clinical data extracted from the electronic medical records of Seoul St. Mary’s Hospital, Korea. Patients who underwent HSCT between January 2013 and December 2018 and tested negative for hepatitis B surface antigen (HBsAg) before undergoing HSCT were included. The associations between HBsAg-RS and demographic information, baseline hepatitis B serological markers, and vaccination status were statistically analyzed.

**Results:**

This study included 1,344 patients, of whom 83.3% tested positive for the hepatitis B surface antibody (HBsAb) during HSCT. HBsAg-RS occurred in 2.2% of HBsAb-negative patients and 3.0% of HBsAb-positive patients, indicating no significant difference in reactivation rates according to HBsAb status. However, positivity for hepatitis B core antibody (HBcAb) was significantly associated with hepatitis B reactivation (HBsAg-RS rate: 8.0%). The vaccination rates were highest in patients who were negative for both HBsAb and HBcAb and had a transient protective effect.

**Conclusion:**

The sufficient patient population enabled the identification of an association between baseline HBcAb positivity and the development of HBsAg-RS. Further prospective studies are warranted to determine optimal vaccination strategies for preventing HBsAg-RS.

## Introduction

Hematopoietic stem cell transplantation (HSCT) is a pivotal treatment option for various hematological malignancies and disorders, offering the possibility of a cure or significant life extension. The procedure involves the removal of the recipient’s bone marrow, typically through high-dose chemotherapy or radiation therapy, followed by infusion of hematopoietic stem cells to restore bone marrow function. Although HSCT can be lifesaving, it causes severe immunosuppression during the early post-transplantation period. During this period, patients are highly susceptible to infections, which can complicate recovery and affect overall survival [1]. Among the associated risks, hepatitis B infection (whether newly acquired or reactivated) poses a serious threat owing to its potential to cause severe liver complications, including cirrhosis and liver failure [2–4].

Hepatitis B surface antigen reverse seroconversion (HBsAg-RS) was recently identified in a previously seronegative patient. By conventional definitions, this result alone does not indicate the development of clinically significant active hepatitis but can serve as an alarming indicator that warrants monitoring for the potential development of active hepatitis. In the context of immunosuppression following HSCT, viral reactivation is common in patients with pre-existing infections, including those with HBsAg-RS, and the associated risk factors are well established [5–7]. However, consensus is lacking regarding the management of a broader spectrum of conditions that can lead to HBsAg-RS, including de novo infections and other causes [8–14]. Therefore, the optimization of assessment methods for HBsAg-RS, the identification of relevant key influencing factors, and the accumulation of information would significantly enhance the management of hepatitis B after HSCT.

In this study, we retrospectively reviewed the medical records from a single center to provide basic and exploratory information on the incidence of and factors influencing HBsAg-RS. We primarily focused on hepatitis B surface antibody (HBsAb) and hepatitis B core antibody (HBcAb), which are influential factors in the development of active hepatitis B in immunosuppressed patients, and analyzed their association with HBsAg-RS. Additionally, we examined the changes in both factors over time after HSCT to determine whether the risk varied depending on the test results at different time points. In terms of prophylactic measures, we determined how vaccination practices varied according to patients’ underlying conditions and analyzed the association between vaccination and HBsAg-RS. Through this approach, we aimed to validate our findings against existing knowledge on preventing the development of active hepatitis in immunosuppressed patients and propose directions for future research.

## Materials and methods

### Data source and ethics

The data used in this study were extracted from the electronic medical record registry of The Catholic University of Korea Seoul St. Mary’s Hospital, Seoul, Korea. The patient’s baseline demographic information, such as age and sex, and the clinical factors, such as mortality status at the last follow-up, date of transplantation, serial results of hepatitis B virus (HBV) serological markers (HBsAg, HBsAb, and HBcAb), and date and number of hepatitis B vaccinations received, were obtained. The study was conducted in accordance with the guidelines of the Declaration of Helsinki and approved by the Institutional Review Board of Seoul St. Mary’s Hospital (approval number: KC23RISI0329). Given that this study exclusively utilized publicly available, anonymized, and de-identified data, the requirement for informed consent was waived.

### Study population and design

Patients who underwent HSCT at the Department of Hematology, Seoul St. Mary’s Hospital between January 2013 and December 2018 were included in the analysis, while those who tested positive for HBsAg at the time of HSCT were excluded. HBsAg-positive findings within 3 months after HSCT were not classified as HBsAg-RS as they were attributed to direct transmission from the donor and were therefore excluded from the analysis. Patients who died within 6 months after HSCT were also excluded from the analysis. Therefore, the final cohort included patients who tested negative for HBsAg before HSCT and who survived for at least 6 months after transplantation.

### Definition

In this study, an HBsAb titer level of less than 10 IU/L was classified as HBsAb negative, while a titer level of > 10 IU/L was classified as HBsAb positive. The baseline HBsAb status referred to the titer measured at the time of HSCT. The 3-month and 6-month HBsAb levels referred to the titers measured at 3 and 6 months after transplantation, respectively. HBsAg-RS was defined as the seroconversion from a negative HBsAg test result before HSCT to a positive HBsAg test result after HSCT. Patients were considered vaccinated if they received at least one dose of the hepatitis B vaccine after HSCT. Hepatitis B vaccine was identified using the following codes: DV-HBBJ1, DV-HBEF1, DV-HBEF1-E, DV-HBEJ0.5, DV-HBEJ0.5-E, DV-HBEJ0.5-H, DV-HBEJ0.5-K, DV-HBEJ0.5-T, DV-HBEJ1, DV-HBEJ1-E, DV-HBEJ1-H, DV-HBEJ1-K, DV-HBEJ1-T, DV-HBG0.5-T DV-HBG1-T, DV-HBGF0.5, DV-HBGF0.5-E, DV-HBGF1, DV-HBGF1-E, DV-HBGF1-T, DV-HBGJ0.5, DV-HBGJ0.5-E, DV-HBGJ0.5-H, DV-HBGJ0.5-K, DV-HBGJ0.5-T, DV-HBGJ0.5T1, DV-HBGJ1, DV-HBGJ1-E, DV-HBGJ1-H, DV-HBGJ1-K, T, DV-HBG1-T, DV-HBGF0.5, DV-HBGF0.5-E, DV-HBGF1, DVHBGF1-E, DV-HBGF1-T, DV-HBGJ0.5, DV-HBGJ0.5-E, DV-HBGJ0.5-H, DV-HBGJ0.5-K, DV-HBGJ0.5-T, DV-HBGJ0.5T1, DV-HBGJ1, DV-HBGJ1-E, DV-HBGJ1-H, and DV-HBGJ1-K.

### Statistical analysis

Demographic characteristics, vaccination status, and index year of HBV markers were presented using descriptive statistics, with each outcome summarized as the number of patients (*N*) and corresponding frequencies (%). The differences in vaccination practices according to baseline serological markers were evaluated using the Fisher’s exact test. The associations between HBsAb, HBcAb, and hepatitis B vaccination status and the incidence of HBsAg-RS were assessed using Fisher’s exact test to determine their potential as a predictor of HBsAg-RS. At this stage, the HBsAb levels at the time of HSCT and 3 and 6 months after HSCT were also considered. If evidence suggested that the HBsAb level could influence the occurrence of HBsAg-RS, statistical analyses, such as multivariate Cox regression analysis, were performed to measure the protective effect. Additionally, the cumulative incidence of HBsAg-RS over time was analyzed using the Fine-Gray sub-distribution hazard model, with mortality considered as a competing risk. Statistical analyses were performed using the R software (version 4.2.3). Statistical significance was determined as a *p*-value of less than 0.05.

## Results

### Patient characteristics

**Fig. 1:**
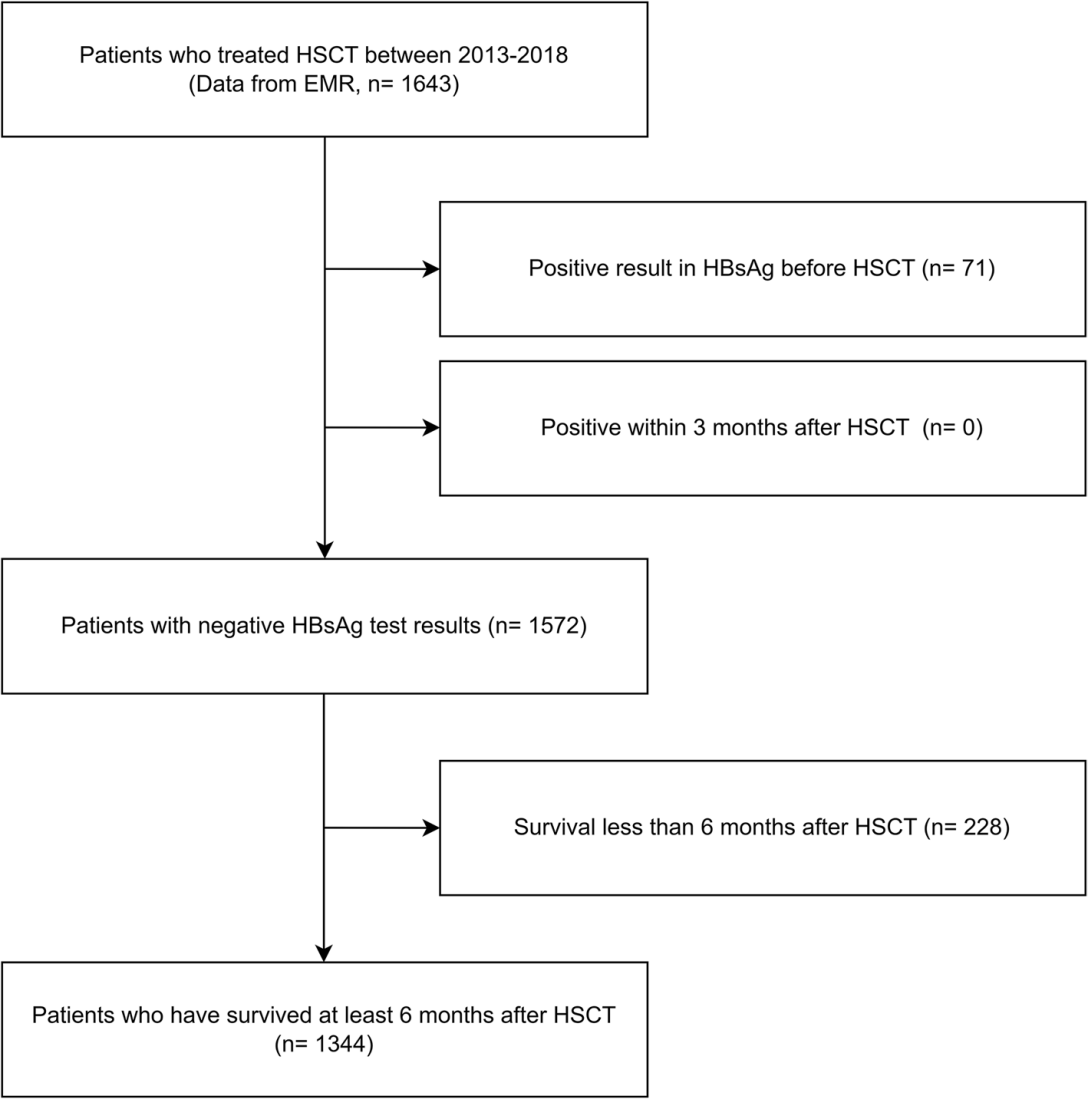
Flow diagram of the participant selection process. Abbreviations: HBsAb, hepatitis B surface antibody; HSCT, hematopoietic stem cell transplantation; EMR, electronic medical record

Of the 1,643 patients who underwent HSCT between 2013 and 2018, 1,344 who were HBsAg negative before HSCT and survived for at least 6 months after transplantation were included in the final cohort and were eligible for analysis. Figure 1 shows a flow diagram of the participant selection process. Among these patients, 1,119 (83.3%) were HBsAb positive and 225 (16.7%) were HBsAb negative at the time of HSCT. Of the 1,344 patients, 711 (52.9%) were HBcAb negative, 376 (28.0%) were HBcAb positive, and 257 (19.1%) were not tested for HBcAbs at the time of HSCT. The distribution of these serological markers was consistent across the index years. In all serological marker groups, men outnumbered women, with a median age of 45 years. Notably, the HBsAb-positive group had a higher proportion of women compared with the baseline HBsAb-negative group (*p* = 0.015), and their mean age was 3 years older (*p* = 0.014). Although the sex distribution was similar between the HBcAb-positive and HBcAb-negative groups, the HBcAb-positive group had a significantly older mean age (13 years, *p* < 0.001). The baseline characteristics of the patients are summarized in Table 1. The most common clinical condition in the final cohort was acute myeloid leukemia (40.9%), followed by acute lymphoblastic leukemia (22.6%) and myelodysplastic syndromes (14.4%) (Table 2).

**Table 1:** Baseline characteristics of the study population

Variables	Total (*n* = 1,344)	Baseline HBsAb			Baseline HBcAb			
		**Negative** **(*n* = 225)**	**Positive** **(*n* = 1,119)**	***p*-value**	**Negative** **(*n* = 711)**	**Positive** **(*n* = 376)**	**NA** **(*n* = 257)**	***p*-value**
**Sex**				0.015				0.482
Male, n (%)	741 (55.1)	141 (62.7)	600 (53.6)		381 (53.6)	210 (55.9)	150 (58.4)	
Female, n (%)	603 (44.9)	84 (37.3)	519 (46.4)		330 (46.4)	166 (44.1)	107 (41.6)	
**Age**, median (Q1–Q3)	45 (33–54.3)	43 (30–53)	46 (33–55)	0.014	40 (29–51)	53 (46–58)	41 (32–52)	< 0.001
**Hepatitis B vaccination**				< 0.001				< 0.001
NOT vaccinated, n (%)	821 (61.1)	105 (46.7)	716 (64.0)		369 (51.9)	300 (79.8)	152 (59.1)	
Vaccinated, n (%)	523 (38.9)	120 (53.3)	403 (36.0)		342 (48.1)	76 (20.2)	105 (40.9)	
**Index year**								
2013, n (%)	214 (15.9)	25 (11.1)	189 (16.9)		61 (8.6)	42 (11.2)	111 (43.2)	
2014, n (%)	217 (16.1)	31 (13.8)	186 (16.6)		86 (12.1)	51 (13.6)	80 (31.1)	
2015, n (%)	212 (15.8)	34 (15.1)	178 (15.9)		95 (13.4)	61 (16.2)	56 (21.8)	
2016, n (%)	231 (17.2)	55 (24.4)	176 (15.7)		146 (20.5)	75 (19.9)	10 (3.9)	
2017, n (%)	224 (16.7)	42 (18.7)	182 (16.3)		157 (22.1)	67 (17.8)	0	
2018, n (%)	246 (18.3)	38 (16.9)	208 (18.6)		166 (23.3)	80 (21.3)	0	

*Abbreviations: NA* missing results, *n* Number, *HBsAb* hepatitis B surface antibody, *HBcAb* hepatitis B core antibody, *Q1* first quartile, *Q3* third quartile

**Table 2:** Disease characteristics of the study population

Disease	Total (*n* = 1,344)	HBsAb		HBcAb		
		**Negative**	**Positive**	**Negative**	**Positive**	**NA**
ALL	304 (22.6%)	62 (20.4%)	242 (79.6%)	178 (58.6%)	69 (22.7%)	57 (18.8%)
AML	550 (40.9%)	62 (11.3%)	488 (88.7%)	331 (60.2%)	203 (36.9%)	16 (2.9%)
BPDCN	2 (0.1%)	1 (50.0%)	1 (50.0%)	2 (100.0%)		
CLL	1 (0.1%)		1 (100.0%)	1 (100.0%)		
CML	19 (1.4%)	4 (21.1%)	15 (78.9%)	8 (42.1%)	6 (31.6%)	5 (26.3%)
CMMoL	10 (0.7%)	3 (30.0%)	7 (70.0%)	4 (40.0%)	3 (30.0%)	3 (30.0%)
ESRD	3 (0.2%)		3 (100.0%)			3 (100.0%)
HLH	1 (0.1%)		1 (100.0%)			1 (100.0%)
HL	7 (0.5%)	3 (42.9%)	4 (57.1%)	4 (57.1%)	1 (14.3%)	2 (28.6%)
HGG	1 (0.1%)		1 (100.0%)		1 (100.0%)	
MDS	193 (14.4%)	33 (17.1%)	160 (82.9%)	64 (33.2%)	41 (21.2%)	88 (45.6%)
MM	22 (1.6%)	11 (50.0%)	11 (50.0%)	15 (68.2%)	2 (9.1%)	5 (22.7%)
MPAL	3 (0.2%)	2 (66.7%)	1 (33.3%)	3 (100.0%)		
MPN	28 (2.1%)	8 (28.6%)	20 (71.4%)	8 (28.6%)	14 (50.0%)	6 (21.4%)
NHL	68 (5.1%)	16 (23.5%)	52 (76.5%)	38 (55.9%)	17 (25.0%)	13 (19.1%)
PNH	3 (0.2%)	1 (33.3%)	2 (66.7%)			3 (100.0%)
SAA	118 (8.8%)	18 (15.3%)	100 (84.7%)	47 (39.8%)	17 (14.4%)	54 (45.8%)
SCA	5 (0.4%)	1 (20.0%)	4 (80.0%)	4 (80.0%)		1 (20.0%)
Thalassemia	5 (0.4%)		5 (100.0%)	4 (80/0%)	1 (20.0%)	
Atypical CML	1 (0.1%)		1 (100.0%)		1 (100.0%)	

*Abbreviations:* *HBsAb* hepatitis B surface antibody, *HBcAb* hepatitis B core antibody, *NA* missing results, *n* number, *ALL* acute lymphoblastic leukemia, *AML* acute myeloid leukemia, *BPDCN* blastic plasmacytoid dendritic cell neoplasm, *CLL* chronic lymphocytic leukemia, *CML* chronic myeloid leukemia, *CMMoL* chronic myelomonocytic leukemia, *ESRD* end-stage renal disease, *HLH* hemophagocytic lymphohistiocytosis, *HL* Hodgkin’s lymphoma, *HGG* hypogammaglobulinemia, *MDS* myelodysplastic syndromes, *MM* multiple myeloma, *MPAL* mixed phenotype acute leukemia, *MPN* myeloproliferative neoplasms, *NHL* non-Hodgkin’s lymphoma, *PNH* paroxysmal nocturnal hemoglobinuria, *SAA* severe aplastic anemia, *SCA* sickle cell anemia, *Atypical CML* atypical chronic myeloid leukemia

### Hepatitis B vaccination rate

The vaccination rate among the HBsAb-positive group was 36.0%, which was lower than that among the HBsAb-negative group (53.3%; *p* < 0.001). The vaccination rate in the HBcAb-positive group was 20.2%, which was significantly lower than that in the HBcAb-negative group (48.1%, *p* < 0.001) (Table 1). In the HBsAb-positive group, the vaccination rate of HBcAb-negative patients (46.0%) was significantly higher than that of the HBcAb-positive patients (19.2%; *p* < 0.001). In the HBcAb-negative group, the vaccination rate of HBsAb-negative patients (55.2%) was significantly higher than that of HBsAb-positive patients (46.0%; *p* = 0.041) (Table 3). A similar trend was observed in the HBcAb-positive group; however, the difference was not significant.

**Table 3:** Hepatitis B vaccination status according to baseline HBsAb and HBcAb status

		**Baseline HBsAb**		***p*-value**
		**Positive**	**Negative**	
**Baseline HBcAb**	**Positive**	68/354 (19.2%)	8/22 (36.4%)	0.095
	**Negative**	251/546 (46.0%)	91/165 (55.2%)	0.041
***p*-value**		< 0.001	0.114	

*Abbreviations:* *HBsAb* hepatitis B surface antibody, *HBcAb* hepatitis B core antibody

### Association of serological markers, vaccination status, and HBsAg-RS incidence

Approximately 3% of the patients developed HBsAg-RS. Among the serological markers, HBcAb was the only marker significantly associated with the development of HBsAg-RS (*p* < 0.001). HBsAg-RS occurred in 8% of the patients with positive baseline HBcAb test results. Although the incidence of HBsAg-RS was lower in vaccinated patients than in non-vaccinated patients, the difference was not significant (*p* = 0.509), suggesting that the vaccine did not contribute significantly to the prevention of HBsAg-RS (Table 4). Based on the results of this analysis, we stratified the groups according to the baseline HBcAb levels to determine the relationship between HBsAb levels at 3 and 6 months post-HSCT and the occurrence of HBsAg-RS; however, the results were not significant (Table 5).

**Table 4:** Incidence of reverse seroconversion according to HBsAb, HBcAb, and vaccination status

		**Baseline HBsAb**			**Baseline HBcAb**			**Anti-hepatitis B vaccination**		
		**Negative** **(*n* = 225)**	**Positive (*n* = 1,119)**	***p*-value**	**Negative** **(*n* = 711)**	**Positive** **(*n* = 376)**	***p*-value**	**Not vaccinated** **(*n* = 821)**	**Vaccinated** **(*n* = 523)**	***p*-value**
**Reverse seroconversion**	**Did not occur**	220 (97.8%)	1085 (97.0%)	0.664	708 (99.6%)	346 (92.0%)	< 0.001	795 (96.8%)	510 (97.5%)	0.509
	**Occurred**	5 (2.2%)	34 (3.0%)		3 (0.4%)	30 (8.0%)		26 (3.2%)	13 (2.5%)	

*Abbreviations:* *HBV* hepatitis B virus, *HBsAb* hepatitis B surface antibody, *HBcAb* hepatitis B core antibody

**Table 5:** Reverse seroconversion rates according to HBsAb status at 0, 3, and 6 months after HSCT

	**Baseline HBsAb**			**3-month HBsAb**			**6-month HBsAb**		
	**Negative**	**Positive**	***p*-value**	**Negative**	**Positive**	***p-value***	**Negative**	**Positive**	***p-value***
**All patients**	5/225 (2.2%)	34/1,119 (3.0%)	0.664	1/8 (12.5%)	28/879 (3.2%)	0.234	0/6 (0.0%)	12/363 (3.3%)	1.000
**HBcAb negative**	0/165 (0.0%)	3/546 (0.5%)	1.000	0/7 (0.0%)	3/487 (0.6%)	1.000	0/4 (0.0%)	0/170 (0.0%)	1.000
**HBcAb positive**	4/22 (18.2%)	26/354 (7.3%)	0.087	1/1 (100.0%)	22/260 (8.5%)	0.088	0/2 (0.0%)	10/102 (9.8%)	1.000

*Abbreviations: HBV* hepatitis B virus, *HBsAb* hepatitis B surface antibody, *HBcAb* hepatitis B core antibody, *HSCT* hematopoietic stem cell transplantation

**Fig. 2:**
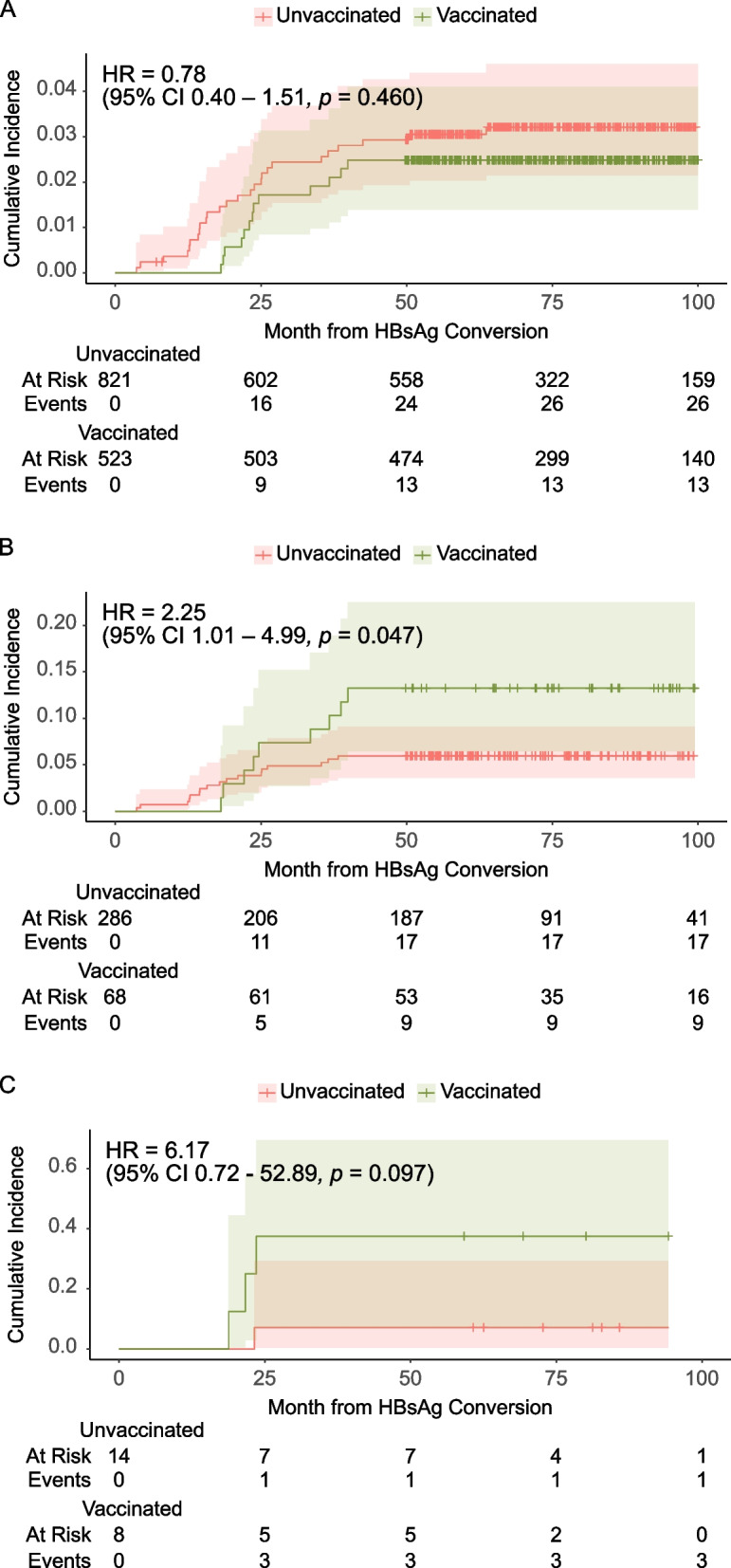
Cumulative incidence of reverse seroconversion after HSCT in unvaccinated (red line) and vaccinated (green line) patients: (A) All patients, (B) HBcAb-positive and HBsAb-positive patients, and (C) HBcAb-positive and HBsAb-negative patients. Abbreviations: HBV, hepatitis B virus; HBsAb, hepatitis B surface antibody; HBcAb, hepatitis B core antibody; HSCT, hematopoietic stem cell transplantation; HR, hazard ratio; CI, confidence interval

In the baseline HBcAb-positive group, the incidence of HBsAg-RS was higher in patients with negative baseline and 3-month HBsAb levels. To obtain more precise information, a cumulative incidence analysis was performed (Fig. 2). The results indicated that when all patients were considered, the risk of HBsAg-RS did not differ according to vaccination status (hazard ratio [HR] = 0.78, *p* = 0.460). However, vaccinated patients who tested positive for HBcAb at baseline tended to be at greater risk of HBsAg-RS than their counterparts, regardless of the baseline of their HBsAb levels (HR = 2.25, HBsAb positive; HR = 6.17, HBsAb negative).

### Disposition and protective effect of HBsAb

Among the patients with a negative baseline HBsAb test result, 61.7% (74/120) seroconverted to positive 3 months after HSCT. However, at six months, the positivity rate among these patients decreased to 27.5%, indicating that the antibodies did not persist over time. Further analysis of the patients with baseline HBsAb-positive test results revealed that the median baseline HBsAb level was 90.5 mIU/mL (range: 11.9–1000) in the HBsAg-RS ( +) group. The median HBsAb level increased to 102.8 mIU/mL (range: 13.0–839.5) after 3 months and further increased to 110.9 mIU/mL (range: 55.14–230.2) after 6 months. The median baseline HBsAb level in the HBsAg-RS( −) group was 97.9 mIU/mL (range: 2.7–1,000). The median HBsAb level in this group significantly increased to 174.6 mIU/mL (range: 3.48–1,000) at 3 months, followed by a slight decline to 126.6 mIU/mL (range: 0–1000) at 6 months. Overall, the HBsAg-RS( −) group exhibited higher median HBsAb levels at 3 and 6 months compared with the HBsAg-RS( +) group.

A multivariate Cox regression analysis was conducted to evaluate the potential protective effect of HBsAb levels against the development of HBsAg-RS, considering the differences in HBsAb levels between the HBsAg-RS outcome groups. The analysis demonstrated that the baseline HBsAb levels were not significantly associated with the risk of developing HBsAg-RS (HR = 1.003, p = 0.077). Similarly, the HBsAb levels measured 3 months after HSCT were not significant predictors of HBsAg-RS occurrence (HR = 0.997, *p* = 0.381), and the association remained nonsignificant at 6 months (HR = 0.997, *p* = 0.507). Additionally, no other factors provided meaningful protective effects.

## Discussion

This single-center study examined the changes in several indicators related to HBV activation after HSCT in correlation with baseline serological markers and vaccination status. The primary concern was whether immunosuppressants used to prevent transplant rejection impacted the ability of vaccination to produce HBV antibodies and whether the tendency for HBV activation varied based on the status of baseline serologic markers. The baseline positivity rates for HBsAg, HBsAb, and HBcAb were consistent with those reported in previous studies conducted in Korea, indicating that the dataset’s composition was appropriate for analysis [15–17]. However, the low incidence of HBsAg-RS limited the number of patients available for analysis, making it difficult to draw definitive conclusions. Nevertheless, a few exploratory considerations can be identified for managing the risk of active hepatitis following HSCT.

Current guidelines for managing hepatitis B reactivation in immunosuppressed patients recommend a tailored approach based on baseline serologic markers [14]. According to these guidelines, HBcAb-positive patients should be initially assessed for the risk of developing active hepatitis. Subsequently, prophylactic antiviral therapy is recommended for high-risk individuals, while close monitoring is recommended for low-risk individuals. Therefore, vaccination is not the preferred management approach for HBcAb-positive patients. In the HBcAb-negative group, no specific measures are required for HBsAb-positive patients, while vaccination is recommended exclusively for HBsAb-negative patients. These findings align with the results of our study, which demonstrated that vaccination rates were highest among the HBcAb-negative and HBsAb-negative patients. Thus, vaccination aligns with the guidelines for conventionally immunosuppressed patients. The comparable vaccination levels observed in patients negative for both markers and the traditionally indicated population suggests the implementation of a more conservative prophylactic approach.

Baseline HBcAb-positive patients exhibited the highest incidence of HBsAg-RS, making HBcAb the only significant factor associated with the occurrence of HBsAg-RS. This finding suggests that the reactivation of HBV, rather than a new infection, is more likely the cause of HBsAg-RS after HSCT. In this study, neither transient HBsAb positivity nor vaccination had a significant protective effect on HBcAb-positive patients, as confirmed by the analysis of HBsAb positivity rates by time point and cumulative incidence. These results underscore the need for more intensive prophylaxis or close monitoring, rather than vaccination, in patients with positive baseline HBcAb test results [14, 18].

The vaccine is typically administered approximately 1 year after HSCT, leading to the speculation that patients with HBsAb seroconversion observed 3 months after HSCT may have acquired HBsAb directly from the donor. However, the rapid decline in the antibody positivity rate within 3 months suggests that this early seroconversion likely had minimal impact on reducing the risk of HBsAg-RS. Although vaccination appears to confer partial protective utility, the cumulative incidence analysis demonstrated that vaccinated patients, regardless of their HBcAb status, had a significantly lower incidence of HBsAg-RS (up to approximately 24 months after HSCT) compared with unvaccinated patients. Further studies are required to determine the most effective method for vaccinating HBsAb-negative patients.

This study has several limitations that should be considered when interpreting the results. The retrospective design and single-center data collection may limit the generalizability of the results. Additionally, the lack of data on the exact timing of vaccination in relation to HSCT and the lack of detailed information on the dose and type of vaccine administered may have affected the results.

In conclusion, our findings advocate a personalized approach to managing the risk of HBsAg-RS in HSCT recipients, emphasizing the need for targeted prophylaxis in high-risk individuals based on their HBcAb status. Future studies should validate these observations in larger, multicenter cohorts and explore the mechanisms underlying the differential responses to vaccination, ultimately guiding the development of more effective management protocols for HBV in the setting of HSCT.

## Data Availability

Owing to the nature of the hospital data, the original datasets cannot be provided; however, the analyzed results are available upon request from the corresponding author.

## Data availability

No datasets were generated or analysed during the current study.
