# Correction: Hepatitis B surface antigen reverse seroconversion after hematopoietic stem cell transplantation according to the baseline serological marker levels and vaccination status: a single‑center database analysis

**DOI:** 10.1007/s44313-024-00043-5

**Published:** 2024-11-13

**Authors:** Soo Young Kang, Heejoo Ko, Raeseok Lee, Sung‑Soo Park, Seunghoon Han

**Affiliations:** 1 Department of Pharmacology, College of Medicine, The Catholic University of Korea, Seoul, Republic of Korea; 2 Division of Infectious Diseases, Department of Internal Medicine, College of Medicine, The Catholic University of Korea, Seoul, Republic of Korea; 3 Division of Hematology, Seoul St. Mary’s Hematology Hospital, College of Medicine, The Catholic University of Korea, Seoul, Republic of Korea; 4 Department of Clinical Pharmacology and Therapeutics, Seoul St. Mary’s Hospital, College of Medicine, The Catholic University of Korea, Seoul, Republic of Korea

**Correction** **:** **Blood Res 59, 31 (2024)**

**https://doi.org/10.1007/s44313-024-00035-5**

Following publication of this article [1], it has come to our attention that the abbreviation for hepatitis B surface antibody appeared incorrectly as HBsAg on two occasions in the captions of Table 4 and 5.

The correct abbreviation should have been HBsAb.

The original article has been updated.

## References

[1] Kang SY, Ko H, Lee R, et al. Hepatitis B surface antigen reverse seroconversion after hematopoietic stem cell transplantation according to the baseline serological marker levels and vaccination status: a single‑center database analysis. Blood Res. 2024;59:31. 10.1007/s44313-024-00035-5. 39412690 10.1007/s44313-024-00035-5 PMC11485279

